# We Remember… Elders’ Memories and Perceptions of Sleeping Sickness Control Interventions in West Nile, Uganda

**DOI:** 10.1371/journal.pntd.0004745

**Published:** 2016-06-02

**Authors:** Vanja Kovacic, Inaki Tirados, Johan Esterhuizen, Clement T. N. Mangwiro, Michael J. Lehane, Stephen J. Torr, Helen Smith

**Affiliations:** 1 Department of Vector Biology, Liverpool School of Tropical Medicine, Liverpool, United Kingdom; 2 Department of Animal Science, Bindura University of Science Education, Bindura, Zimbabwe; 3 Warwick Medical School, University of Warwick, Coventry, United Kingdom; 4 Centre for Maternal and Newborn Health, Department of International Public Health, Liverpool School of Tropical Medicine, Liverpool, United Kingdom; KARI-Trypanosomiasis Res Centre, KENYA

## Abstract

The traditional role of African elders and their connection with the community make them important stakeholders in community-based disease control programmes. We explored elders’ memories related to interventions against sleeping sickness to assess whether or not past interventions created any trauma which might hamper future control operations. Using a qualitative research framework, we conducted and analysed twenty-four in-depth interviews with Lugbara elders from north-western Uganda. Participants were selected from the villages inside and outside known historical sleeping sickness foci. Elders’ memories ranged from examinations of lymph nodes conducted in colonial times to more recent active screening and treatment campaigns. Some negative memories dating from the 1990s were associated with diagnostic procedures, treatment duration and treatment side effects, and were combined with memories of negative impacts related to sleeping sickness epidemics particularly in HAT foci. More positive observations from the recent treatment campaigns were reported, especially improvements in treatment. Sleeping sickness interventions in our research area did not create any permanent traumatic memories, but memories remained flexible and open to change. This study however identified that details related to medical procedures can remain captured in a community’s collective memory for decades. We recommend more emphasis on communication between disease control programme planners and communities using detailed and transparent information distribution, which is not one directional but rather a dialogue between both parties.

## Author Summary

African elders are recognized by their communities as important traditional leaders. This role gives them an influential position, which is commonly overlooked by disease control programmes. We focused on sleeping sickness a disease which has a long history of control interventions in our study location in north-western Uganda. We interviewed elders to explore their memories of past interventions. This is important because negative perceptions of past interventions could influence how communities perceive control programmes today. Interviewed elders described sleeping sickness control interventions dating from the 1960s and more recent interventions from 1990s. Invasive diagnostic procedures, toxic side effects of treatment and long hospitalization were remembered from the later interventions. Despite these negative experiences, elders, however, observed recent improvements in treatment and had no negative perceptions of sleeping sickness control programmes. We conclude that community experience with control programmes remains in memories for decades, and we recommend the involvement of elders in planning of these interventions. This would be particularly beneficial because they are aware of the historical contexts of disease control in their environment, have insights into socio-cultural aspects of their communities and may serve as spokespersons between beneficiary community and programme implementers.

## Introduction

All cultures around the world promote a normative respect for their elderly populations. Elders are often sought for their advice on life wisdoms in a rapidly changing world [1]. In addition, African elders have always been awarded with authoritative roles as leaders in social affairs, mediators in disputes, marriages, funerals and rites of passage rituals. Elders in traditional African societies have also been respected as agents possessing supernatural powers, manifested as links with ancestors’ spirits, and in healing and fortune telling skills [2–4].

This special role makes African elders influential agents in rural communities. In a rapidly changing African social and physical environment, they serve as a link between past and future and are therefore sometimes recognized as essential partners in health programmes. In Uganda, for instance, the UN liaised with local elders in efforts against female genital circumcision [5]. In Kenya, elders equipped with mobile phones contributed significantly towards the documentation of new-born birth weight [6]. There are also numerous studies reporting an impact of elders, who serve as guardians of HIV positive orphans, on management of paediatric HIV (for instance [7]).

Senior members of a community also act as a living memory of the past. An oral tradition is still a widely used way of preserving knowledge and cultural identity in rural Africa. The richness of this source has been recognized and documented in the context of historical- and development-oriented research [8–10], however this approach has seldom been used in disease control programmes. Exploring the collective memory of communities that participated in disease control programmes is especially insightful in cases of prolonged and potentially traumatic interventions.

One such example is sleeping sickness (human African trypanosomiasis; HAT), a parasitic disease, caused by trypanosomes and transmitted by tsetse flies (*Glossina*). There are two forms of HAT: the acute *rhodesiense* form occurs in south east Uganda; the second type is the slowly developing *gambiense* form, which is endemic in the north west of Uganda, where this study was conducted. Both forms in the initial stage (first stage) of disease cause symptoms such as headache, fevers, and general fatigue, but then progress to a second stage where neurological symptoms such as aggression, delirium, hallucinations and disturbed sleeping patterns may be exhibited. Due to these unique manifestations, there is clear historical documentation of the disease occurring in Africa, during the time of the slave trade, along with information on how patients were treated. Captured slaves, for instance, who exhibited the signs and symptoms of sleeping sickness were whipped for their “laziness*”* [11]. Furthermore colonial texts documented the imposition of numerous methods of sleeping sickness control measures on local African populations. In Uganda, these methods included establishment of check points and control of human migration in and out of sleeping sickness areas; treatment with toxic experimental drugs, such as the arsenic based atoxyl and strychnine; formation of segregated treatment camps [12, 13] and forced vegetation clearance along rivers to control tsetse [14]. The territory was strictly administered and penalties were imposed for those who failed to respect the rules [15].

The last major HAT epidemic in the history of Uganda was attributed to the influx and re-settlement of refugees from infected areas of South Sudan back into northern Uganda in the 1990s [16, 17]. After Uganda achieved independence in 1962, sleeping sickness epidemics were mostly controlled by international NGOs, such as Médecines sans Frontières (MSF), which in collaboration with the local governments established active screening campaigns in affected areas and treated patients in the local hospitals [18, 19]. No records, however, exist on how communities reacted to these interventions. Although communities in Uganda witnessed a number of HAT epidemics and were involved in numerous control programs, there is little attention in the literature to their perspectives. By examining elders’ memories in this study we aimed to explore what memories have been preserved in relation to colonial HAT control measures and more recent interventions implemented by MSF. We are not aware of any other attempt to document collective memory of HAT affected communities in Uganda. The study is especially relevant in the context of new diagnostic tools and treatment of HAT, which are expected to be introduced in the next couple of years [20–22]. Understanding of the community experiences of previous programs may greatly impact on how these new tools are accepted and utilized.

The main objectives of this study were to i) evaluate what experience is preserved in the memories of elders in relation to sleeping sickness; ii) assess if any of the memories are associated with collective trauma and; iii) determine if past trauma can be neutralized by more positive experiences with interventions against HAT. With this study we aimed to contribute towards better communication between providers and beneficiaries of HAT control programs and ultimately to support HAT elimination efforts.

## Methods

### Ethics approval

The study protocol and procedures for obtaining participants’ consents were approved by the Research Ethics Committee of Liverpool School of Tropical Medicine (ref: 11.73) and Uganda National Council of Science and Technology (UNCST) Ethics Committee (ref: SS-2561). Local district and sub-county administrative authorities and village chiefs were informed about the study and their permission sought prior to data collection. All participants were informed about the study, and encouraged to ask questions; their voluntary participation and right to withdraw from the study were emphasised and their written consent was obtained. In case of illiterate participants a fingerprint was collected in front of a literate witness. Consent was also obtained for the use of photo materials. Signed or finger-printed consent forms are stored securely at the offices of the LSTM tsetse research project, Arua, Uganda.

### Study area

Despite a decrease in the number of new HAT cases globally, 459 of cases of *gambiense* HAT were reported from Uganda between 2008 and 2012 [23] and West Nile still represents an important focus in efforts to eliminate HAT [24]. MSF-France, which responded to the last major HAT epidemic in West Nile in the 1990s, established the treatment centre in Omugo [18] which is still one of the main facilities in the region for diagnosis and treatment of HAT. Village and sub-county health centres and district hospitals are referral points for diagnosed or suspected HAT cases.

The study was conducted between July 2011 and March 2012 in rural areas of Arua and Maracha Districts (within the coordinates: 03°09’27.92”-03°12’16.57”N, 30°51’06.00”-30°55’01.68”E). Districts in Uganda are composed of sub-counties, which are organizational units joining several villages. The study villages were purposively selected based on their location in relation to known local HAT foci. Four villages were selected in an area where, in 2010 (i.e. within 12 months of the study) Médecins sans Frontières (MSF) conducted active screening and still detected HAT cases (HAT foci; HAT+). The other four villages were selected from an area where, based on medical records, no HAT cases have been reported prior to MSF campaigns (non-HAT foci; HAT-) and have therefore been excluded from active screening [19]. These villages were selected to compare if community memories of HAT and control interventions differ from those with more recent experiences with screening and treatment campaigns.

The predominant ethnic group in this area is Lugbara and main religious orientations are Christian (Roman Catholic and Protestant) and Muslim. Communities gain their livelihoods through small scale farming. They mostly plant food crops such as cassava, beans, maize and sweet potatoes and breed goats, cattle and sporadically pigs. Tobacco is planted in some areas as a cash crop. The structure of the villages is arranged in traditional household units with older and younger generations of relatives living in separate huts and sharing the same compound.

### Data collection

In-depth interviews were conducted face-to-face with 24 elders from eight villages in their homes. The interviews were conducted by the corresponding author (VK; PhD; medical anthropologist; female) and a Ugandan female interpreter. None of the participants were fluent in English; all interviews were therefore conducted in the local language (Lugbara) by directly translating questions posed by VK and answers provided by participants back to English by interpreter. The interpreter received training in interviewing and interpretation techniques prior to the data collection process.

Participants were selected using the snowball sampling technique [25]: starting with an initial small sample of elders in each location, we subsequently asked elders to identify others who may be willing to participate in interviews. This approach led us from one elder to another in each village and finally we interviewed eight women and fifteen men until we reached the data saturation point [25]. The selection criteria was not based on participants’ age but on communities’ own recognition of their members as ‘elders’, to ensure that the definition of ‘elder’ corresponded with community understanding of this role. The average reported age of the participants was 71 years; however most of them were unable to state their exact year of birth. Interviews were conducted using an interview guide (S1 Text) with several general open-ended questions, and probes to obtain further details on their memories of HAT and disease control interventions. The interview guide was pretested; questions and probes were discussed and adjusted with interpreter to obtain the closest interpretation to the original meaning of the questions. All discussions were recorded on a digital voice recorder and VK wrote down the field notes. After each interview the corresponding author and interpreter conducted a debriefing session to compare observations they obtained during the interviewing sessions and discuss data saturation.

### Data analysis

Audio files were later transferred to a computer and transcribed into Word documents by the interpreter, supervised by the corresponding author. All transcripts were read several times by VK in order to develop codes. Maxqda software [26] was used for coding transcripts and organizing them into a matrix, consisting of an Excel spreadsheet with quotes corresponding to codes. Each sheet was then analysed and validated by VK and HS using a thematic analysis approach to identify main themes and describe differences between participants from HAT and non-HAT foci. The most commonly reported patterns of opinions, as well as opposing views, were identified in the process.

## Results

Six main themes were identified in the analysis of the interviews with participants, and each is discussed under a separate sub-heading. Participant quotes are used to illustrate the meaning of each theme (Boxes 1–6).

### Disease associated with examination of neck glands

**Fig 1:**
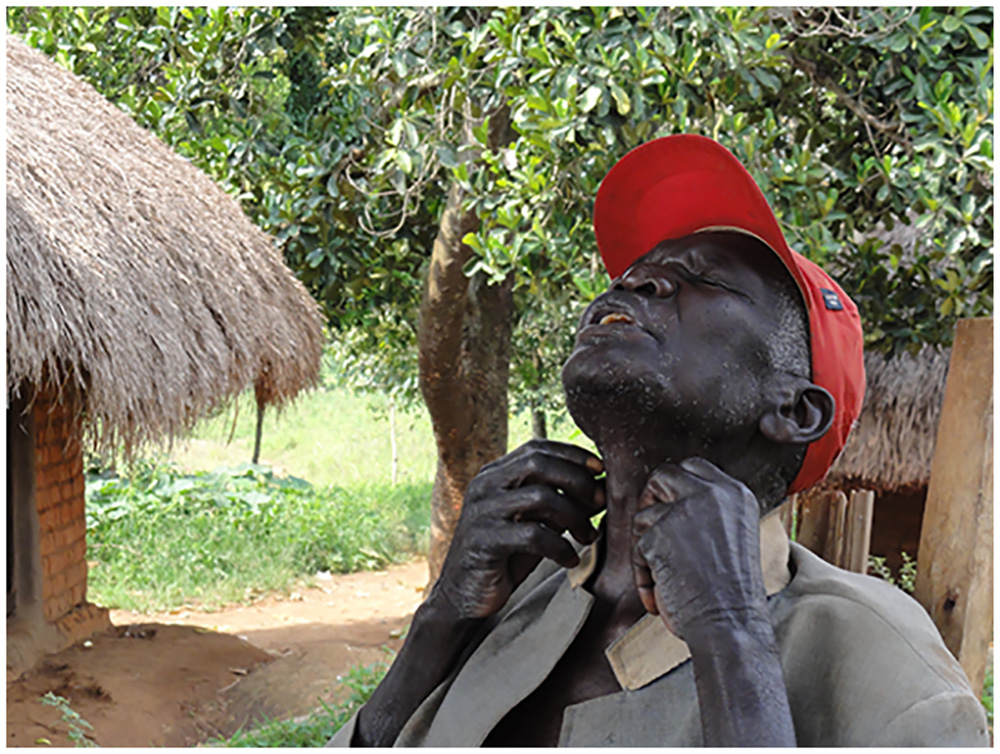
An elder gesturing examination of neck lymph nodes. Palpation of lymph nodes on each side of the neck was the only HAT diagnostic measure in colonial times; these HAT medical campaigns were remembered by interviewed elders.

As soon as sleeping sickness was mentioned to the participants in relation to the past, many of them lifted their hands to the side of their necks, their gestures indicating examination of lymph nodes (Fig 1). Regardless of the location of the village in HAT-foci (HAT+) or non-HAT foci (HAT-), all participants remembered neck examination and many of them participated in this type of testing (box 1). These memories are drawn from the early 1940s to 1960s period, when most of them were children but some from the younger generation referred to the stories they heard from their parents. Mostly, they did not know at the time what disease they were tested for and indicated that they were informed about sleeping sickness when ‘whites [white foreigners] arrived in the area. Some mentioned that the disease was called ‘gland’ and one participant thought they were testing two different diseases: ‘gland’ and ‘sleeping sickness’.

Elders (male and female participants) from both areas (HAT+ and HAT-) had vivid memories of the severity of sleeping sickness and many remembered the last epidemic in the 1990s and attributed it to the migration of refugees from South Sudan. In contrast, participants who do not live in a current HAT focus (HAT-) reported that HAT had not been a major problem for them, even in the past.

**Box 1. Disease associated with examination of neck glands**

*“This disease started a long time [ago] and many people died of it*. *(*…*)*. *They did not know what was happening to people*, *and they didn’t know what kind of disease [it was] since they [people] lived in remote areas*. *And this was before the whites [white foreigners] came to this area*, *so they [people] didn’t have the knowledge”*

(Elder 7, male, HAT+).

*“When your neck was checked that time*, *they would get [diagnose] two things; [first] one*, *they would find that you have sleeping sickness and the second [one] would be called gland whatever it meant”*

(Elder 17, male, HAT+).

“They would say they were checking the neck-it was the disease of the neck; but we didn’t know the name”

(Elder 24, female, HAT-).

“In the old days they used to check neck and it was called ‘gland’ which now is called sleeping sickness”

(Elder 21, male, HAT+).

*“People brought this disease from Sudan*, *to where they escaped during liberation war [as refugees]*

(Elder 16, male, HAT+).

*“No*, *not many cases [of HAT] were here in our area”*

(Elder 1, male, HAT-).

### Sleeping sickness has been associated with traditional causes of disease

Both male and female participants, but only from HAT+ areas, described how sleeping sickness was associated with traditional causes of disease. Some commented that this association was common before health information was distributed by foreigners. Other participants however, indicated that HAT symptoms were associated with traditional causes in recent times as well. Describing, for instance, treatment choices of their sick relatives, diagnosed for HAT in the 1990s, they explained, that both: biomedical and traditional treatment was used. HAT symptoms were interpreted as traditional disease after medical treatment, which was used as the first option, failed to improve health of their relatives. Two traditional categories of disease were mentioned in relation to HAT: ‘witchcraft’ and ‘poison’. Witchcraft was associated with social tensions within the clan and capacity of elders to ‘curse the person and bring sickness over them’. Sacrifice of a goat and sharing meat with the elder, identified as causative agent, was described as a way to ‘settle the matter’, by one participant. ‘Poison’ was described as an evil deed of somebody ‘ill wishing’. Participants explained that ‘poison’ can be diagnosed and treated by traditional healers who ‘wash the patient’ with engine oil to confirm traditional diagnoses and then treat them by traditional means.

**Box 2. Community associated sleeping sickness with traditional causes of disease**

*“This was before people like you (white foreigners) came up to us*. *Earlier*, *in my grannies times*, *when people got sick*, *people didn’t believe in natural death*. *Those people who were elderly*, *they had to have a say (in the community); they could curse another person (and bring sickness)*. *In this case people needed to look for a goat*, *they came (to the elder) and slaughtered a goat*, *so that this elder could eat*, *and then forgive them (for mistake that make him curse them)*. *There should be settlement of the matter and forgiveness takes place*. *That is what would take the sickness away*. *And those days when the person was caught by sleeping sickness*, *the people would say it is witchcraft”*

(Elder 13, male, HAT+).

*“He (sick relative) was taken to Omugo (treatment centre)*. *They treated him*, *he came back as though he was ok*. *But later we discovered that his body was swelling-expanding in size*, *and I thought this was witchcraft practice in Lugbara culture against him*. *Someone must have done something on him”*

(Elder 15, male, HAT+).

*“When the sickness threw him (down) here (at home)*, *we thought it was poison*. *(…)*. *But some people said*, *maybe it would be good to take him to the hospital*. *Then they (hospital health workers) found it was that disease (sleeping sickness)*. *But we are saying that he died of poison”*

(Elder 14, female, HAT+).

*“It’s a poison that killed my husband after all*. *What happened to him before his death was*, *that sickness attacked him (…)*, *manifesting as a headache*. *The sickness intensified by throwing him down (he collapsed)*. *We tested for the poison in the body*, *and they (traditional healers) found it was poison (…)*. *The person (traditional healer) who washes (for poison) gets the roots of a tree*, *and he mixes it with engine oil*, *then he/she washes you with it*. *If it washes smoothly*, *then there is no poison*, *but if it gets stuck to your skin*, *then there is poison*. *He (my husband) died after being washed (traditional ritual was performed)*. *They (healers) gave him treatment for poison*, *but it didn’t help” *

(Elder 14, female, HAT+).

*“It’s after you tried all these other medical drugs on yourself in vain (one would think of traditional treatment)*. *For example you are taken to hospital and they test you for malaria*, *it’s not there (not diagnosed positive)*. *Then they (health workers) test for some other diseases*, *and they don’t find them*, *so they transfer you from maybe Arua hospital to Kuluva*. *(…) Even there they try on you some other drugs*, *but all those things all (of them)-antibiotics (too) will fail*. *Then maybe even some medical personnel can tell you*:*-Maybe you need to check with people at home*, *you may need to clear some issues at home (indicating that sickness is caused by traditional cases)*. *That’s when traditional healer comes to wash you now (perform traditional diagnostic ritual)”*

(Elder 14, female, HAT+).

### Recall of colonial medical interventions: Fear in children, but not in adults

The elders, from both areas (HAT+ and HAT-) recalled that mobilisation for ‘gland examination’ happened once a year and was carried out by medical teams who moved from parish to parish. Villagers were informed about the time and place of gathering by government representatives. Participants commented that testing was compulsory and the entire village, including children, gathered. Families lined up for neck examination, their names were called, and the sides of their necks checked manually. Participants recalled that positive cases were separated from negative ones. Those who were young children at the time or were not diagnosed as positive had no recollection as to what happened with the positive group. Most of the participants from non-HAT foci speculated that positive cases were injected on the spot and then sent home. In HAT foci some participants remembered a treatment camp where there were houses built of clay and where people were treated (injected) at the same site where they were examined (box 3). Most participants recalled that in recent times, patients were transferred to Arua hospital.

According to both, male and female participants, sleeping sickness testing was mandatory. In cases of non-compliance, there were different sanctions in place including interrogation, imprisonment, levy of fines and physical punishment. Despite these disciplinary measures, the vast majority from both areas reported that they willingly participated; they did not report or remember any fears associated with testing or treatment. Positive attitudes towards examination were also associated with the non-invasiveness of the neck examination which did not require collection of blood samples. They associated the examination with offers of help, also a positive association and perception. Some participants reported their childhood memories as being associated with fear of white foreigners and injections, but none of them reported their parents being fearful. Many participants remembered that the fear was not of examination but rather that the fear among adults was that they would get the disease and not be able to receive treatment.

**Box 3. Recall of colonial medical interventions: fear in children, but not in adults**

*“They [positive cases] remained in Chilio [where neck examination was happening] and there were structures of camps raised to admit them in (*…*)*.*They were built like these grass thatched houses and many in numbers [that's how a treatment camp looked like]”*

(Elder 21, male, HAT+).

*“If someone refused to go [for testing]*, *after the exercise [testing] ended*, *they would send a sub-parish chief to ask*, *to question the person*. *Because sub-county chief had a list of people and he would see who did not go*…*They would question that person- why he didn’t go*? *And they would fine him*, *and the fine would be*…*Maybe you would need to pay a goat”*

(Elder 7, male, HAT-).

*“Eaah*! *It would be so bad if someone refused to go for gland examination*! *If you didn’t go*, *they would say you have brought the disease in the place*, *so you were taken to prison*. *They would assume that you are the one bringing more infection or disease in the area”*

(Elder 16, male, HAT+).

“*And if you rebelled against the message [about testing]*, *they would come to get you and take you [away] to be caned [beaten by stick]”*

(Elder 21, male, HAT+).

*“Those were whites [who were involved in neck examination]; so we [children] wondered*: *are they coming to eat us*? *We feared*, *we didn’t know the reason [for them being there]*, *so we feared*. *(*…*)*. *In our thinking was*, *maybe they will even retain us there; why are they calling us there*? *And the colour*, *they are people of different race [white]*, *they are different*. *So that was the essence of fear [for us*, *children]*”

(Elder 13, male, HAT+).

*“Only the kids of my age at the time would run away [were afraid of neck examination] (*…*)*. *What were you afraid of as a kid was just the injections*. *Syringe entering into your body*. *I didn’t like that*. *If you are a child and you see that blood [with injections] you cry so much*. *You don’t like any injection in your body”*

(Elder 3, male, HAT-).

*“People from the village didn’t fear [neck examination]*. *The fear was of not [getting] tested*! *It was better to get tested*…*They were saying that there is a disease which has come*. *People didn’t know which disease*, *but they wanted to be tested”*

(Elder 2, male, HAT-).

*“Since testing was meant to help being cured*, *nobody feared”*

(Elder 19, female, HAT+).

### Reluctance and fear associated with more recent medical interventions

Participants who were not from a HAT focus stated that after the period of ‘gland examination’, no more medical interventions related to HAT were carried out in their area. Thus, only memories from inhabitants living in HAT foci are reported. Participants remembered that after independence in 1962, there was a period without any intervention until MSF France started their campaign in the late 1980s and 1990s at the peak of the epidemic with active village-based screening and treatment of patients in regional hospitals. The organization of active screening and spread of information was in elders’ memories, which echoed those of earlier times. Village chiefs were involved in informing the village members and they ensured that villagers were present at the screening (box 4).

Both male and female elders reported that the use of new medical approaches created some reluctance among community members. At the time, this reluctance was associated with the length of treatment, side-effects of treatment and drug resistance, and high patient mortality. One participant was convinced that in the old days, people could live with HAT and it would just ‘make them dull’, but that once treatment was provided, the disease ‘changed form’ and caused people to die. Many negative associations were also related to diagnostic procedures, such as collection of blood samples and lumbar puncture and rumours associated with them that caused suspicion. Blood collected during diagnostic procedures, for instance, was believed to be collected and sold for transfusion purposes. One participant told of a rumour that the medical team was believed to be involved in the release of tsetse in order to gather people for testing bloods; then they could use the blood collected to sell it. However, despite these complaints, many of the elders reported that most of the community still participated in active screening.

**Box 4. Reluctance and fear associated with more recent medical interventions**

*“What I remember is that community participated in work when the health centre coordinated [and announced] that they are coming on such and such day for testing*. *So the information is sent to us as the government representatives*. *Then for us we would take the information to churches*, *schools where people gather*, *and then we would announce whereby such a day*, *the community would gather*, *the congregation would be there”*

(Elder 14, female, HAT+).

*“And in those earlier times [n the 1990s during MSF France treatment campaign] you were kept long [in the hospital]*. *And for you to get well*, *it may take six months”*

(Elder 14, female, HAT+).

*“What scared the people is that*: *the treatment*, *the help [they received]*, *it was not good (*…*)*. *Sometimes you grow fat [you swell]*, *when given the medicine*. *Yes*. *You grow fat*! *And then you became normal [again after side effects are over]”*

(Elder 10, male, HAT+).

*“That treatment was not all that good*. *For example*, *if I got that disease [HAT]*, *it [the disease] could come back again [after treatment in relapsing cases]”*

(Elder 10, male, HAT+).

*“The people were admitted to the hospital and next time they would be brought back [to the village] as corpses*. *So that created fear in people”*

(Elder 14, female, HAT+).

*“During earlier times people used to not to die from that disease; they used to die when they were old; it would just make them to be dull [mentally compromised]*, *but you would die at the old age*. *But now*, *recently*, *when they started giving drugs*, *the disease changed the form*. *It is making people mental and then they die”*

(Elder 13, male, HAT+).

*“They take blood from here [points at the finger]*, *put it in machine and the worm [parasite] will appear there*. *But if they don’t get it here*, *then they would come and take blood from here [pointing at the upper hand] (*…*)*. *They remove big amount of blood*. *People feared because (…) they were saying that people taking blood [medical team]*, *they will go and sell it “*

(Elder 13, male, HAT+).

OPPOSING VIEW:

*“Those who were ignorant had many stories [rumours about testing]*. *But eventually many people come for testing”*

(Elder 12, male, HAT+).

### New treatment approaches and increased knowledge improved community perceptions

The second medical campaign, carried out by MSF Spain (2010–2011) is remembered differently. Elders form HAT focus areas (HAT+) where MSF campaigns were carried out noticed improvements in treatment, which seemed to increase community trust and reduce general reluctance towards medical interventions (box 5). Particularly male participants noticed that durations of hospitalization were shorter, there were no relapse cases, treatment had fewer side-effects and that no deaths occurred in the process. The new treatment regime, i.e. Nifurtimox / Eflornitine combination therapy, was indeed introduced in the second MSF campaign [19]. Most participants declared that the community response to testing was better during this campaign, and also that fewer cases were diagnosed compared to the previous intervention period. They also reported that there were attitudinal changes within the community itself, having already been sensitized in previous campaigns. Also, many participants had personally witnessed development of the disease in their family members and were therefore aware of the importance of testing and treatment. In general, participants reported that negative associations and fear were lower during the last treatment campaign.

**Box 5. New treatment approaches and increased knowledge improved community perceptions**

*“And when the first group came [MSF France] and you were taken to Omugo [hospital]*, *the duration [of treatment] was long- two weeks*. *But with the second group*, *recently*, *the duration [of treatment at the hospital] is three to four days and then you are back”*

(Elder 10, male, HAT+).

*“But today*, *if you are treated*, *disease will not come back again (*…*)*. *In the recent treatment [campaign]*, *the same people [who were tested and treated years ago] were found with disease again*. *That one [information] tells me that the medicine they were using first is not the same they are using now”*

(Elder 10, male, HAT+).

“*They are all right [all the members of community who were diagnosed]; no cases of death [occurred with the Spanish section of MSF] (*…*)*.*These days it [HAT] is treated easily*, *it is treated easily*! *Because if you are treated you will be all right”*

(Elder 10, male, HAT+).

*“The [community] response was better [when MSF Spain arrived]*! *Because people have already known about the disease*, *some have lost their dear ones*. *They got to know that disease needs to be treated”*

(Elder 12, male, HAT+).

“The fears have reduced and also the rate of death [due to HAT] is slowing [is reduced]”

(Elder 14, female, HAT+).

### Previous organized tsetse control programmes were not recalled as imposed

Only participants from the current HAT foci remembered organized activities related to tsetse control. Both male and female participants remembered cutting vegetation of the river banks—an activity which was organized from the 1950s until Ugandan independence (1962). Male participants remembered more details about these activities. Some of them reported that their parents or relatives participated in land clearance. Clearance of vegetation along river banks was first organized as forced voluntary labour, but was later transformed into paid employment. Re-settlement from the river banks to higher level sites in the village was also reported, but one of the elders commented that this was done willingly by community members, who were aware of the seriousness of the disease and also not concerned about the land ownership ‘since the land was free’. During this time, information about the link between tsetse and sleeping sickness started to spread within the community. Elders also commented that vegetation clearance significantly contributed towards control of sleeping sickness at the time, and so there appeared to be no negative associations with these interventions.

**Box 6. Previous organized tsetse control programmes were not recalled as imposed**

*“The work [of vegetation clearance] was given in hands of local government representatives*. *So they would just get people in the village*, *whether you are willing or not*, *they put you in front to do the work; whoever you are*, *whatever your status*, *as long as you are in that village”*

(Elder 13, male, HAT+).

*“Vegetation clearance started as voluntary*, *but there were some people who were weak while others were able-bodied; the weak began to withdraw [from the programme] and some people were sick to do the work*. *So the white- District Commissioner suggested that*, *it was better if they would employ people who were able bodied to do this work as their daily routine*. *So they started to get paid*, *and the voluntary work was left out (*…*)*. *The payment was six cents per month”*

(Elder 16, male, HAT+).

*“They discovered this disease*, *and government came in to organize the slashing [clearing of vegetation]; the people who were living along the river side*, *whose houses were along the river side*, *were transferred away*, *they were brought instead up here [on the hill away from the river] (*…*)*. *People did not complain [about being resettled] since the disease was real*. *During that time*, *land was free*, *people were using it [the lower parts next to the river] for cultivation and grazing of the animals; nobody would complain”*

(Elder 16, male, HAT+).

“They [people involved in slashing] were informed that they were slashing to keep off the tsetse flies that spread sleeping sickness”

(Elder 17, male, HAT+).

*“It was clean*…*When they maintained the river banks it maintained the rivers clean and this disease called sleeping sickness disappeared at that time”*

(Elder 11, male, HAT+).

## Discussion

Elders’ memories, regardless of the location of their village (in or out of the local HAT focus) spanned from examination of lymph nodes in colonial times. More recent active screening and treatment campaigns were remembered only in the villages in HAT focus, which indicates that information about disease control interventions remains localized. Some negative memories dating back to the 1990s were associated with diagnostic procedures, treatment duration and treatment side effects. These memories, however, were combined with memories of the negative impacts related to sleeping sickness epidemics, particularly in HAT foci. More positive associations and memories from the recent treatment campaigns were reported, especially because of their observations regarding improvements in treatment. Similarly, no negative memories were associated with past tsetse control interventions.

This study showed that memories of the elders are well preserved. This is supported by other studies that demonstrated how oral traditions and historical narratives, which are strongly present across the African continent, are passed from generation to generation for several decades [27–29]. It is important to acknowledge that information stored in collective memory influences community responses to disease control programs. A recent study of barriers to HAT testing in DRC [30], for instance, revealed that memories of the previous interventions, particularly prohibitions after treatment, hampered community participation at current active screening. The imposed restrictions, communicated by health workers in the past, aimed to manage adverse effects of treatment with the toxic melarsoprol. This information, however, created taboos, which have been preserved despite the introduction of new drugs for the treatment of second-stage of gambiense-HAT.

On some occasions, community reactions to disease control programs are linked with memories of other, non-related events. One such example is related to Ebola crises in Liberia [31], where a community associated a curfew imposed by the government during the latest Ebola epidemic with the civil war. This caused additional fear and reluctance to adhere to control measures. Hence, careful examination of the context related to both current and past community experiences is needed before programs are implemented.

Our study, however, showed that previous negative memories, were neutralized with more recent positive experiences with HAT interventions, such as the improvements of treatment. This suggests that community attitudes towards disease control programs are prone to change if an opportunity for more positive experiences is provided. An example of positive community attitudes towards tsetse control traps was recorded in the study in West Nile [32]. High acceptance of traps in this study, was related to sufficient information received at the beginning of the intervention and long exposure to traps, which were introduced to the area a decade ago. Community attitudes in these villages were different to the villages in proximity, which were not exposed to the traps previously. When villagers noticed traps deployed during the study and without being informed about their purpose, this caused associations of traps with supernatural powers and fear among community members.

Thus, the assumption that community memory will adapt to the changes in a disease control program merely through observation and without dissemination of information, is unrealistic. The beneficiary community should be the first stakeholder to be informed about the changes occurring in global HAT control strategies, hence transparent communication and frequent dialogue is necessary not only for keeping all the information updated, but also to prevent future negative experience with disease control programs. As shown in our study, negative experiences will remain a part of the collective memory for a long time.

Effective communication with the host communities could be facilitated through engagement of elders and other traditional leaders. Elders, from our observation, are still perceived as authority in rural Uganda. They can therefore break barriers and act as a bridge between program implementers and beneficiary communities. One successful example of the benefit of involving traditional leaders is in HIV/AIDS prevention in Zimbabwe [33]. In this example leaders encouraged behavioural change related to harmful traditional marriage practices which reduced spread of HIV in their communities. Another example, from Botswana, showed how traditional leaders managed to defuse tensions among community members relating to the role of traditional and biomedical practices concerning male circumcision at the beginning of the program. Their initial collaboration, however, turned into resistance due to miscommunication by program staff and lack of leaders’ participatory involvement [34]. Another example of the failed engagement of leaders is a study on community participation and empowerment in an HIV/AIDS program [35], which showed that a single leader can make the difference between program success and failure. In this HIV/AIDS initiative, the village chief’s beliefs about community values and traditional roles of women completely de-stabilized the program, and as a result, the fundamental program objectives were not achieved. Hence engagement of traditional and other leaders at the beginning of intervention is crucial for successful implementation of disease control programs and their sustainability.

Finally, if disease control programs are not in line with what is acceptable to the beneficiary communities, they will find ways to overcome the obstacles imposed by the program. There is a series of documented evidence on how communities in West Nile rebelled against colonial HAT control measures [36]. The community, for instance, opposed forced re-settlement to HAT free zones and the evacuated people later moved back to their homes [36]. Imposed control in colonial times through a system of permits and passes to limit spread of HAT through human migration, resulted in people traveling through uncontrolled bush tracks. Furthermore, people moved away from water holes and washing places cleared of vegetation to control tsetse, and in their search for privacy started using parts of the river which were left out in the vegetation clearance efforts. Morris [36] suggests that these coercive measures and the responses they promoted from the local communities, served to reinforce the spread of infections rather than preventing the spread of HAT (ibid.).

Some more recent studies from other disease control programs show similar trends. When expectations from the program to control HIV/AIDS [37], for instance, were considered unrealistic by the HIV positive participants in Homa Bay (Kenya) they engaged in a series of different strategies to overcome obstacles. HIV positive patients managed to navigate between expectations of their social environment, which would have been challenged if they strictly followed the rules of the HIV control program and still appeared adherent to the program. The recent Ebola crisis in West Africa also provides examples of mismatch between local realities and imposed control measures resulting in communities rebelling against the rules and for instance hiding their sick relatives [38]. This misunderstanding and related lack of trust ultimately resulted in exacerbation of the epidemic instead of its control [39, 40]. Listening to the voices of beneficiary communities when planning disease control programs is an obvious but, surprisingly, commonly ignored fact.

In summary, disease control programs are often ignorant to the important and insightful position of African elders. Elders in leadership positions are treated as “gate keepers”, and besides seeking access to the populations they are in charge of, little attempt is made to consult them on other matters. Dialogue with elders and other traditional leaders could have a twofold benefit for disease control programmes: firstly, elders could help gain an understanding of any previous negative experiences with disease control which could hamper implementation of disease control programs; and secondly elders could act as active communicators between program implementers and beneficiary communities.

### Limitations

Elders’ memories may have been affected due to age, so recall bias may have occurred when discussing their memories. Only elders who had the ability to articulate their thoughts were interviewed, which means that those that did not, but who may have had thoughts and memories to discuss, were not included in the study.

Biases may also have occurred during direct interpretation from Lugbara to English which was conducted while carrying out interviews and focus group discussions. To minimize this bias, we carefully selected the interpreter based on their previous work experience as a translator and facilitator. A series of training sessions were organized (data collection methods, ethical principles of research, transcription and translation skills) for our research team before the data collection process was begun. Additional quality control on translation was ensured during the transcription process which was carried out by an independent member of the research team fluent in Lugbara and trained in social sciences.

### Conclusions

Consulting elders was a useful framework for capturing collective memories about experiences with past control programs and it reflected current community attitudes towards participation in HAT control. Particularly elders from the villages that experienced more recent HAT control campaigns were knowledgeable about the improvements of treatment in the history of HAT control they witnessed in their villages. We recommend this research framework is used and memories documented before HAT or other community-based interventions are introduced. This will become extremely relevant in the context of introducing new diagnostic and treatment regimens which HAT affected communities are not familiar with.

Furthermore, research in contexts where community trauma is likely to have occurred in the past, such as in early HIV/AIDS interventions or in the recent Ebola crisis in West Africa, may use the same methodological framework with the following steps: i) exploring memories and/or perceptions of past control interventions, ii) acknowledgement, if any, of collective trauma having occurred and iii) discussion with elders and other village leaders on how to prevent potential trauma in the future. We recommend disease control planners to consider the historical context and community perceptions of disease control programs before they launch new disease control interventions.

## Supporting Information

S1 Text Interview topic guide. (DOCX)
