# Supplementary material for: We Remember… Elders’ Memories and Perceptions of Sleeping Sickness Control Interventions in West Nile, Uganda
Source: PLoS Negl Trop Dis. 2016 Jun 2;10(6):e0004745. doi: 10.1371/journal.pntd.0004745 (PMC4890773; doi:10.1371/journal.pntd.0004745)
Supplement: S1 Text — (DOCX) [file pntd.0004745.s001.docx]

**Study: ELDERS ABOUT HISTORY OF SLEEPING SICKNESS AND TSETSE CONTROL INTERVENTIONS**

**Interview topic guide**

**OBJECTIVES:**

- To explore perceptions of HAT burden from the past until today
- To evaluate if community has any potential traumatic memories related to HAT epidemics and past HAT control interventions
- To explore previous interactions/involvements of the community with HAT control interventions
- To determine elders’ views on current/future HAT control

INTRODUCTION

Explain purpose of the study/Clarify definitions: HAT, tsetse control/Recording of interview/Translations/How data will be used/Confidentiality/Written consent form/ask if any clarification needed

1. PERCEPTION OF HAT BURDEN IN THE PAST UNTIL TODAY

- Can you recall the first memory associated with sleeping sickness in your community?

-What is the story associated with it?

-Who was the person involved?

-How did the story end up?

-From which period was this story (how many years/decades back)?

-How did community treat that person?

-What kind of life did that person have?

-Did this affect person’s family?

- Were there many similar examples of this story in that particular period?

1. PAST EXPERIENCE WITH DIAGNOSIS AND TREATMENT

- How were these patients diagnosed?

-Was there active screening happening at the time?

-Who was carrying out active screening?

-Did you ever participate?

-Can you describe how did it look like?

- How was treatment of HAT at the time?

-Did community know about treatment of sleeping sickness?

-How patients who went through treatment described their experiences in the hospital?

-Did many people die (never returned from the hospital, treatment centre)?

3. PAST INVOLVEMENT OF THE COMMUNITY WITH TSETSE CONTROL INTERVENTIONS

- Were there any activities for controlling tsetse flies at the time?

-When is the first time in your memory that you can recall any such activities?

- What were the activities?
- Who was involved in carrying them out?

-Did community participate in tsetse activities?

-Was this paid labour, voluntary work or were they forced to participate?

-What would happen if they would not participate?

-Who organized community for these activities?

-Who from community would participate (man, women, children)

1. FROM PAST UNTIL TODAY

- In relation to people who suffer from HAT, has anything changed from the past?

-What has changed?

-Is diagnosis/treatment different?

-Is life of HAT patients any easier?

-Did community look at HAT patients differently?

- Did tsetse control change?

-What did change?

- Why do you think these changes happen?

CLOSING REMARKS: Thank you/Ask if any questions

*shaded questions will be used as prompts, to help the conversation if the participant does not elaborate in the first open question

Probing questions will be used throughout to elicit more detailed information or to understand better what is being said – for example, can you tell me more about that, why is that, what do you mean by that?
